# Clinical Decision Support to Reduce Opioid Prescriptions for Dental Extractions using SMART on FHIR: Implementation Report

**DOI:** 10.2196/45636

**Published:** 2023-11-07

**Authors:** D Brad Rindal, Dhavan Prasad Pasumarthi, Vijayakumar Thirumalai, Anjali R Truitt, Stephen E Asche, Donald C Worley, Sheryl M Kane, Jan Gryczynski, Shannon G Mitchell

**Affiliations:** 1 HealthPartners Institute Minneapolis, MN United States; 2 Memorial Hermann Health System Houston, TX United States; 3 Friends Research Institute Baltimore, MD United States

**Keywords:** clinical decision support systems, dentistry, analgesics, electronic health records, EHR, algorithm, design, implementation, decision support, development, dentists, pain management, patient care, application, tool, Fast Healthcare Interoperability Resources, FHIR, Substitutable Medical Applications and Reusable Technologies, SMART

## Abstract

**Background:**

Clinical decision support (CDS) has the potential to improve clinical decision-making consistent with evidence-based care. CDS can be designed to save health care providers time and help them provide safe and personalized analgesic prescribing.

**Objective:**

The aim of this report is to describe the development of a CDS system designed to provide dentists with personalized pain management recommendations to reduce opioid prescribing following extractions. The use of CDS is also examined.

**Methods:**

This study was conducted in HealthPartners, which uses an electronic health record (EHR) system that integrates both medical and dental information upon which the CDS application was developed based on SMART (Substitutable Medical Applications and Reusable Technologies) on FHIR (Fast Healthcare Interoperability Resources). The various tools used to bring relevant medical conditions, medications, patient history, and other relevant data into the CDS interface are described. The CDS application runs a drug interaction algorithm developed by our organization and provides patient-specific recommendations. The CDS included access to the state Prescription Monitoring Program database.

**Implementation (Results):**

The pain management CDS was implemented as part of a study examining opioid prescribing among patients undergoing dental extraction procedures from February 17, 2020, to May 14, 2021. Provider-level use of CDS at extraction encounters ranged from 0% to 87.4% with 12.1% of providers opening the CDS for no encounters, 39.4% opening the CDS for 1%-20% of encounters, 36.4% opening it for 21%-50% of encounters, and 12.1% opening it for 51%-87% of encounters.

**Conclusions:**

The pain management CDS is an EHR-embedded, provider-facing tool to help dentists make personalized pain management recommendations following dental extractions. The SMART on FHIR–based pain management CDS adapted well to the point-of-care dental setting and led to the design of a scalable CDS tool that is EHR vendor agnostic.

**Trial Registration:**

ClinicalTrials.gov NCT03584789; https://clinicaltrials.gov/study/NCT03584789

## Introduction

The United States has experienced an epidemic of opioid overdose deaths, with deaths associated with prescription pain relievers of particular concern [1]. Inappropriate prescribing of opioids, heroin use, and the increase in the use of illicitly manufactured fentanyl and its analogues have driven this unprecedented opioid epidemic. Opioid analgesics are among the most frequently prescribed drugs by dentists [2]. An estimated 5 million people undergo third-molar extractions in the United States each year [3]. Evidence shows that exposure to opioid analgesic prescriptions following dental extractions and other procedures is widespread in the United States [4]. Combining a nonsteroidal anti-inflammatory drug with acetaminophen provides a viable and evidence-based pain management alternative to prescription opioids when better pain control is needed [5].

Electronic health records (EHRs) may contain much of the relevant medical history information needed to make appropriate decisions without navigation of multiple screens required to locate desired information. Unfortunately, dental EHR systems are generally not part of the medical EHRs; therefore, dentists rely on the patients to complete a medical history questionnaire. Currently, the information exchange between dentistry and medicine is hampered by a lack of data standards and interoperability between medical and dental EHR systems [6].

Clinical decision support (CDS) has the potential to improve clinical decision-making consistent with evidence-based care [7-9]. CDS can be designed to save providers time and help them provide safe, personalized analgesic prescribing by bringing together relevant medical conditions, current medications, a prior history of substance use, and additional prescribing information from the state prescription drug monitoring program. The problem with integrating siloed yet important health information [6] and the proposed solution of using CDS to integrate this information into one interface serves as the premise for this research study [10].

The objective of this study was to test the efficacy of two interventions (CDS with and without patient education), compared to the treatment-as-usual approach to decrease opioid prescribing for dental extractions. This manuscript adheres to the iCHECK-DH (Guidelines and Checklist for the Reporting on Digital Health Implementations) [11]. The National Institute of Dental and Craniofacial Research of the National Institutes of Health (NIH) funded this project using a cooperative agreement where they provided oversight, coordination, and facilitation. This paper describes the development of a CDS system using HL7 (Health Level 7) FHIR (Fast Healthcare Interoperability Resources) and the SMART (Substitutable Medical Applications and Reusable Technologies) on FHIR framework.

## Methods

### Development of the CDS

The project overview is presented in Multimedia Appendix 1.

The CDS is built on secure, scalable, and EHR-agnostic core design principles using health care systems’ critical IT infrastructure. Point-of-care, real-time patient medical information extraction is key to the CDS system. This allows for accurate personalized recommendations and integration with the EHR in a manner that fits into the clinical workflow without deviating from the standard-of-care process, facilitating improved CDS use with low or no burden on the health care provider. The CDS system used the industry-standard EHR interoperability method, HL7 FHIR, for data formats and the application programming interface (API) for data exchanges and health care IT. The EHR-agnostic third-party application integration method, the “SMART on FHIR” framework, provides secure authentication and authorization management to the application through a valid existing EHR session. The SMART on FHIR framework built on OAuth 2.0 [12] authorization framework provides secure token and code exchanges to establish access to the FHIR resource server for patient medical record data extraction and presents clinical recommendations in a dental provider interface.

The DIODE (“De-Implementing Opioid Use and Implementing Optimal Pain Management Following Dental Extractions” study) CDS tool follows the EHR data governance model to protect the data privacy and data security of patients. The backend database system for the DIODE CDS tool follows the highest industry standards and enterprise access control policies, limiting data access to only authorized IT personnel who are designated for maintaining the systems. The SMART on FHIR–based DIODE CDS application design approach follows EHR access control policies and privacy settings, allowing only authorized individuals with valid HER-authenticated user sessions to access patient records.

### Translating Local Data Into FHIR-Compliant Data

Data access is limited to specific FHIR resources (Table 1) through the OAuth 2.0 authorization scope, and API access expires in a short time for improved data privacy and security. The pain management CDS is only launched from a valid EHR session on specific patient records, based on context provided by the dentist in a secure manner, enabling the pain management CDS as a single sign-on and saving time for the provider.

Since different versions of HL7 FHIR specification have been developed and implemented by EHR vendors, the pain management CDS selected the HL7 FHIR DSTU2 version, which is supported by multiple leading EHR systems.

**Table 1:** FHIR (Fast Healthcare Interoperability Resources) resource and use.

Source	FHIR resource	Pain management CDS^a^ use
1	AllergyIntolerance.Search (DSTU2)	Relevant allergies.
2	Condition.Search (DSTU2)	Relevant medical conditions from the active problem list and diagnosis.
3	MedicationStatement.Search (DSTU2)	Current medications with potential interactions with pain medications commonly prescribed by dentists.
4	Observation.Search (DSTU2)	Nonprescription substances used by the patient.
5	Patient.Read (DSTU2)	Relevant patient demographic information.

^a^CDS: clinical decision support.

### Pain Medication Recommendations (Algorithm Development)

Pertinent patient medical information is extracted from the EHR as FHIR resources. The pain management CDS uses an algorithm developed by our team to identify patient-specific contraindications for pain medications, including opioid medications, nonsteroidal anti-inflammatory drugs, and acetaminophen. It also identifies patient allergies and conditions potentially impacted by these analgesics.

### RxNorm API

RxNav is a service of the National Library of Medicine, which is part of the NIH, an agency of the US Department of Health and Human Services [13]. RxNav is a browser for several drug information sources, including RxNorm, RxTerms, and Medication Reference Terminology (MED-RT). We relied on the RxNorm code system to translate the system-specific drug or medication names into standardized names. These codes are further used to get ingredient-level information using the RxNav API web service [14]. The ingredient-level information is used in a custom-defined algorithm to find the drug-level interactions.

Medication ingredient-level information is incorporated into the RxNorm web service API to convert brand name or generic name drugs from the EHR to normalized RxNorm code details. The medication ingredient-level information is used in an in-house custom-defined algorithm to identify drug-level interactions. The pain management CDS application uses these drug-level interactions to display patient-level personalized pain management recommendations.

### EHR Integration

**Figure 1:**
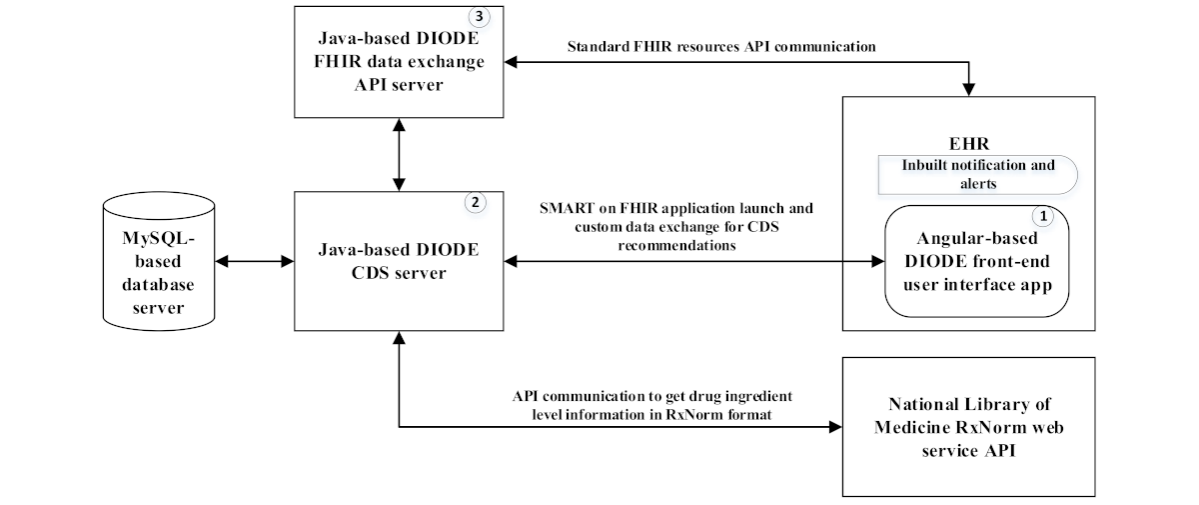
The pain management clinical decision support system (CDS) application architecture. API: application programming; CDS: clinical decision support; DIODE: De-Implementing Opioid Use and Implementing Optimal Pain Management Following Dental Extractions; FHIR: Fast Healthcare Interoperability Resources interface; SMART: Substitutable Medical Applications and Reusable Technologies.

The CDS is designed as a software component-based, reusable subsystem to perform various functions and data flow support. Angular was used as the framework for the user interface, which was embedded into the EHR for integrated workflow support. The Java-based DIODE CDS server is the heart of the system, which performs algorithm processing, data storage, and retrieval from the database server and provides personalized clinical recommendations. The Java-based DIODE FHIR data exchange server facilitates FHIR resource exchanges with the EHR FHIR resource server and provides collected data to the DIODE CDS server. Figure 1 explains the application architecture.

The Angular-based DIODE front-end application was developed for presenting CDS personalized recommendations to an intuitive user interface with subsections for (1) drug interactions, (2) condition considerations, as well as (3) allergies or intolerances and relevant action items. The front-end application is launched from the EHR and communicates with the DIODE CDS server to get the required data to generate the user interface and education materials.

The CDS server is the core of the pain management CDS, which connects to other software components for data extraction and processing, including the management of OAuth 2.0 communication with the EHR authorization server. The CDS server connects to the backend database server for storing patient data extracted from the EHR, which includes personalized recommendation information generated by CDS algorithms. It also retrieves data from the database server to generate the user interface. The FHIR exchange API server communicates with the EHR FHIR resource server to extract patient medical record information in JavaScript Object Notation standard text-based format. The HER’s inbuilt Notification and Alert functionality highlights the CDS prompt in the record when an appropriate clinical condition is met, such as the addition of a tooth extraction procedure to the patient’s chart. By default, the dentist has access to the pain management CDS from the patient header section, and only eligible patient records show the highlighted link to the CDS. A MySQL database server is used to store the CDS data, which includes extracted patient clinical data from the EHR, algorithm mapping data, and generated personalized CDS recommendations.

### Access to the Minnesota Prescription Monitoring Program

The CDS provides a hyperlink to the Minnesota Prescription Monitoring Program for providers to view controlled substance prescriptions from the state-level prescription monitoring database.

### Launching the CDS Application

**Figure 2:**
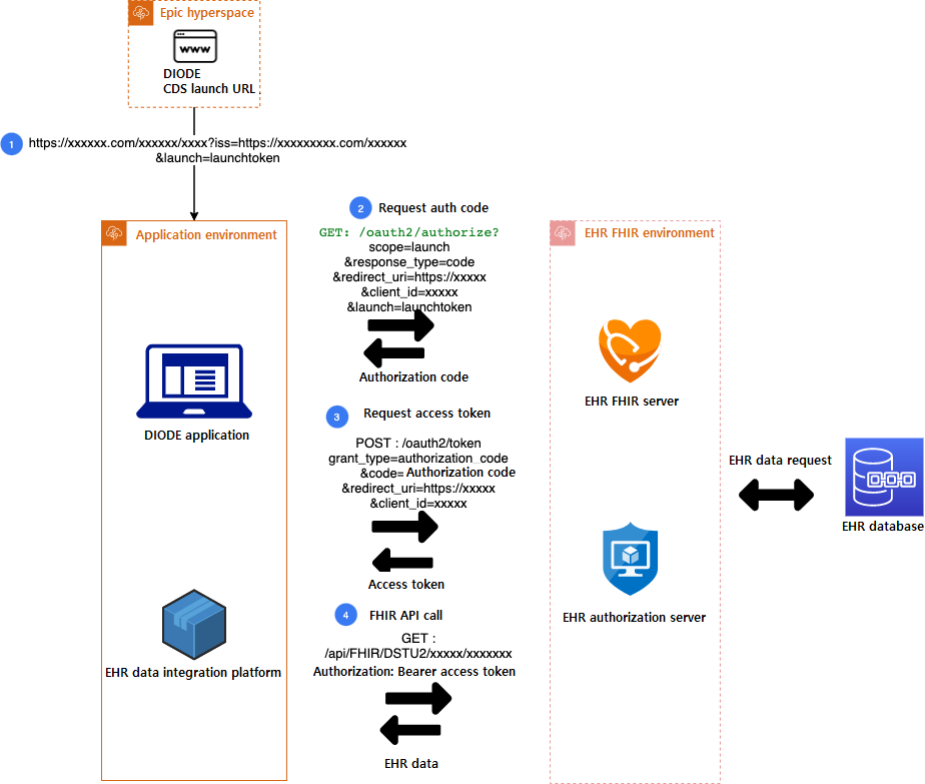
The pain management—OAuth2.0 [12] and the HL7 (Health Level 7) FHIR (Fast Healthcare Interoperability Resources) resource data extraction dataflow diagram. CDS: clinical decision support. DIODE: De-Implementing Opioid Use and Implementing Optimal Pain Management Following Dental Extractions; EHR: electronic health record; FHIR: Fast Healthcare Interoperability Resources.

Since the CDS is embedded in the EHR, the application can be launched, when needed, using a navigation link integrated within the EHR. In the initial launch, the application receives a launch token, which provides a valid EHR user session context and a URL; the URL helps to identify EHR FHIR server information, including EHR authorization server and resource server URL end points. The application receives the authorization code after a successful request along with a launch token. The authorization code prompts an access token, which is used subsequently in multiple FHIR resource API call requests to get the required patient information for CDS use. In the EHR, a header alert is highlighted when patient eligibility criteria are met. The CDS can be launched with 1 click, simplifying navigation to the CDS. Additionally, an HER-specific navigational link was created to access the pain management CDS from any patient chart context. Figure 2 [12] explains the data extraction flow.

### Pain Management CDS Application Interface

**Figure 3:**
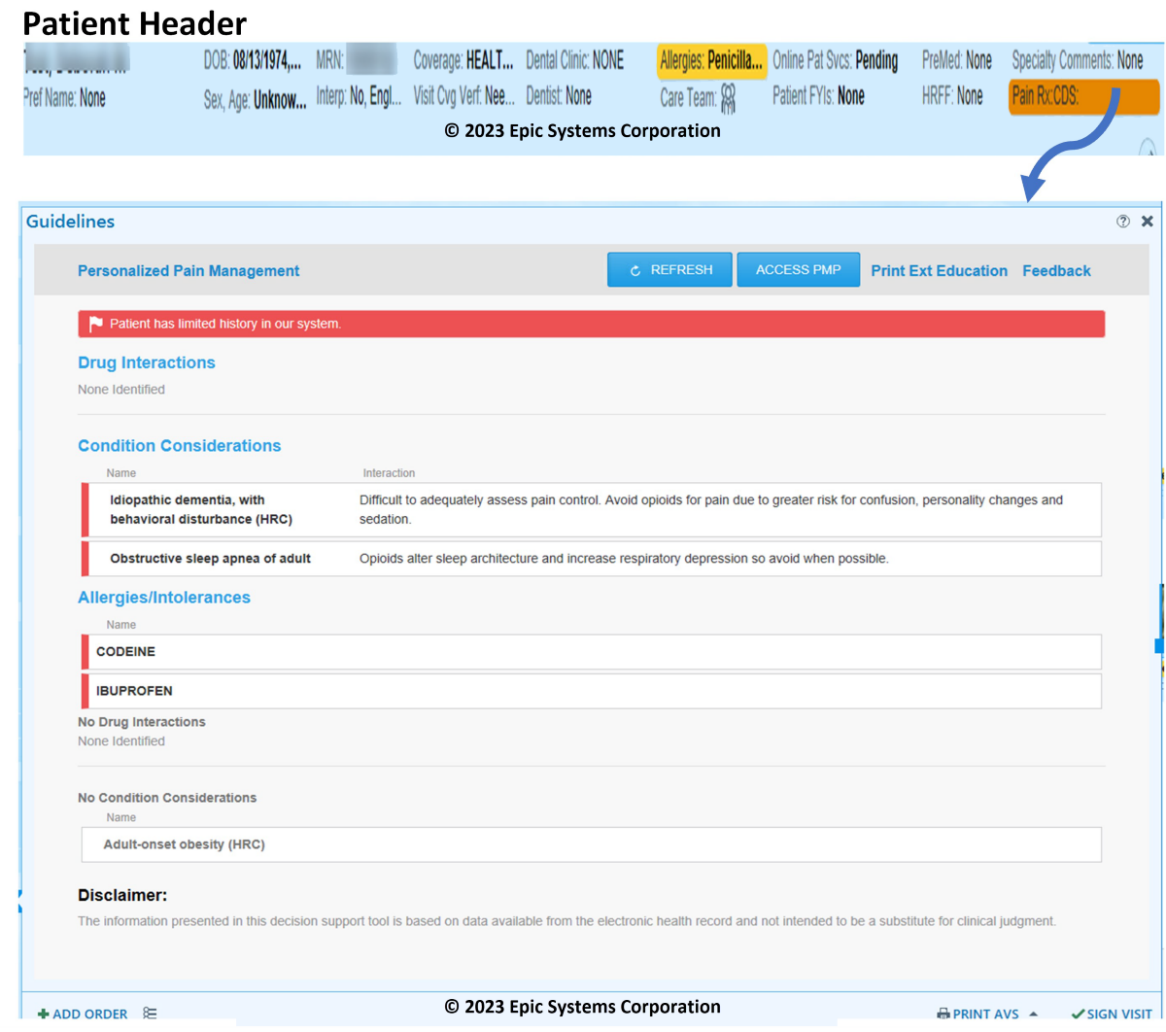
Clinical decision support (CDS) system screen example.

The CDS interface provides a simple summary (Figure 3) of the relevant information that should be considered in deciding about the most appropriate pain management strategy, including the most appropriate analgesic to prescribe or recommend for an individual patient. Relevant information was highlighted in red.

### Addressing Missing HL7 FHIR Resources

The CDS extracts data from the EHR using the HL7 FHIR DSTU2 standard data model, which was lacking a few data items at the time of the CDS design. A few custom data elements were added, such as (1) Patient Pregnancy Status, (2) Patient Breastfeeding Status, and (3) Substance Use Status. These data were extracted using custom functionality provided by the EHR system (Epic Extensions) and sent as part of the SMART on FHIR access token response, which is an extension of standard OAuth 2.0 token response after successful authorization validation.

### Addressing Other Technical Requirements

AngularJS is a JavaScript-based open-source front-end web framework for developing single-page applications. It is maintained mainly by Google and a community of individuals and corporations. It aims to simplify both the development and the testing of such applications by providing a framework for client-side model–view–controller and model–view–view–model architectures, along with components commonly used in web applications and progressive web applications. AngularJS implements the model–view–controller pattern to separate presentation, data, and logic components [15]. Using dependency injection, Angular brings traditionally server-side services, such as view-dependent controllers, to client-side web applications. Consequently, much of the burden on the server can be reduced. AngularJS brings value when dealing with non-Epic systems.

### Statistical Analysis of CDS Use

The CDS was highlighted to alert the dental provider when an extraction was planned but did not open automatically. Therefore, this design offered an opportunity to measure when a clinician opened the CDS, which was an important outcome of CDS use.

Dental provider attributes and the frequency of CDS use are described with counts, percentages, and means (SDs). Differences in CDS use by provider attributes, including provider sex, provider age (<40 vs >40 years), and number of extraction encounters (<100 vs >100) are tested in a generalized linear mixed model (with a logit link and binomial error distribution) containing fixed effects for the provider attributes and a random intercept for the provider to accommodate the clustering of patient encounters within providers. Model-derived percentages and *P* values are presented.

### Implementation (Results)

Among 20 clinics with providers assigned to the intervention arms, 95.0% (19/20) of clinics had at least 1 extraction encounter with the CDS opened. Among 3 oral surgeons assigned to the active intervention arms, 2 opened the CDS for 0.5% (10/1874) of tooth extraction encounters. Among 30 dentists assigned to the active intervention arms, 27 opened the CDS for 24.3% (501/2059) of tooth extraction encounters. Provider-level use of the CDS at extraction encounters ranged from 0% to 87.4%, with 12.1% of providers never opening the CDS (0% of encounters), 39.4% opening the CDS for 1%-20% of encounters, 36.4% opening it for 21%-50% of encounters, and 12.1% opening it for 51%-87% of encounters.

Among 2059 encounters linked to 30 dentists in the active intervention arms, the CDS was opened at similar levels by dentists aged 40 years and younger compared to dentists older than 40 years (27.0% vs 22.5%; *P*=.62). Male and female dentists opened the CDS at similar levels (24.0% vs 26.0%; *P*=.83). Dental providers with 100 or more extraction encounters opened the CDS at similar levels as those with fewer than 100 extraction encounters (26.1% vs 21.5%; *P*=.62).

Among 1061 extraction encounters in the two intervention arms in which an opioid was prescribed, the CDS was opened for 5.4% (57/1061) of encounters. Among 2872 extraction encounters in the two intervention arms in which an opioid was not prescribed, the CDS was opened for 15.8% (454/2872) of encounters.

### Ethical Considerations

The study was approved by the HealthPartners Institutional Review Board (#A17-013). The project was part of a broader quality improvement initiative approved by the HealthPartners Dental Group and did not alter the standard of care for dental extractions; approval by the Dental Group for this minimal risk study was acceptable as an alternative to written informed consent documentation for dentists. Patients on the research exclusion list were not included in the study.

## Discussion

### Principal Findings

This study used a programming interface and integration platform that combines with existing EHRs, patient portals, personal health records, and data warehouses. The 3 key aspects of SMART on FHIR are as follows: (1) a data access layer based on FHIR, combined with a set of constraining profiles that lock down optionality and align vocabularies with Meaningful Use requirements; (2) a security layer that provides narrowly scoped authorization to specific portions of a patient’s record via OAuth 2.0; and (3) a single-sign-on layer using OpenID Connect, which can either integrate with an existing EHR or patient portal session, conveying the current patient, encounter, and other host environment details, or launch independently, such as on a mobile phone or device [12,16,17].

The HL7 FHIR standards are constantly improved, and new data items are added to FHIR resources data model with each new version [18]. Since the custom data extraction varies for each EHR vendor, some data items from the patient’s medical record may not be part of the standard FHIR resources. The custom data elements are sent as part of Access Token Response after successful Authorization providing streamlined data flow without comprising IT security.

The CDS application link is built into the patient header section in the EHR as a clickable link, which can be launched anytime as a SMART on FHIR application by a dentist. The clickable link is highlighted in a yellow background color to get the provider’s attention when an eligible criterion (ie, a tooth extraction procedure) is added to the patient’s chart. This approach is different from the HL7 CDS Hooks approach, which sends the information to the CDS server system on a specific event in the patient’s chart, such as chart open or order entry, and provides a response to show actionable CDS card information.

Overall, results showed that health care providers’ use of the pain management CDS was low and consistent with CDS use in other studies [19]. Postintervention interviews with providers indicated that the app worked exactly as developed and that those who used it regularly found the synthesized health information to be very beneficial, informative, and time saving. Providers who did not use it more than once or twice identified several reasons for not using it consistently. These reasons included the following: forgetting about the CDS, which is plausible, as it was not triggered unless an extraction was treatment planned; some dental providers not modifying their workflow to include checking the CDS; and some finding the visual representation (highlighting in the EHR) to be subtle and easily overlooked in a busy EHR dashboard. Early attempts at opening the CDS that resulted in either problems with functionality or providers determining that the type of information they expected to see was not included also negatively impacted its continued use.

CDS strategies to deimplement opioid prescribing for dental extractions did not lead to reduced opioid prescribing compared to standard practice in the main trial. We found that opioid prescribing declined significantly over time in all conditions. A comprehensive description and results of this clinical trial have been published [20]. HealthPartners [21] was already paying close attention to opioids and undertaking several actions to reduce opioid prescribing by providers while the study was being implemented. In the context of a downward trend in opioid prescribing, dental providers identified several factors that led to reduced reliance on opioids, including governmental and health system opioid prescribing policy changes and the COVID-19 pandemic [22].

We trained providers to open the CDS for all extraction procedures so they could receive a summary of relevant information about potential drug interactions and medical conditions relevant to analgesic prescribing. In qualitative interviews conducted at the end of the study, dentists often described the CDS as something they perceived as useful only if they were considering prescribing an opioid. If another analgesic option was planned or the patient did not have any medical conditions or few current medications, the providers did not see a reason to open the CDS [22]. These findings suggest that the appropriate use of CDS needs to be tailored to more complex or high-risk patient groups. In addition, the training information was sent in an email communication that included training slides and continuing education credit for viewing the slides. We provided contact information for a study team member if there were any questions. CDS was likely negatively impacted by a passive approach to email communication. A more robust in-person or web-based training session for the providers to attend could improve CDS use.

CDS application using the SMART on FHIR framework improves health care interoperability similar to standards applied to other industries [23]. Most EHR databases use a proprietary API. Without tools like SMART on FHIR, it becomes necessary to build a custom connection to each database to access medical data. This is costly, hindering the ability of health care providers and patients to access their data with their preferred technology [24]. SMART on FHIR provides a standard, universal API for accessing the EHR database they use. We estimate that 20% of the budget was used to build and integrate various data elements into the CDS tool. This research project was budgeted at US $500,000 a year and funded for 5 years without an option for additional funding.

### Conclusions

Development of the provider-facing, point-of-care CDS using SMART on FHIR supported the study goals of providing dental practitioners with context-dependent pain management alternatives and enabling patient-centered shared decision-making for pain management. However, CDS use was limited. Strategies to improve its use need to be considered for CDS tools to realize their potential. Future opportunities for CDS use and tools such as SMART on FHIR include clinical topics where provider behavior needs to align with current evidence and would benefit from integrating medical data. Table 2 summarizes key insights and recommendations. This report follows the Implementation Reporting Guidelines.

**Table 2:** Insights and recommendations.

Insight	Recommendation
Well-designed CDS^a^ does not insure utilization.	Engage providers in the design and provide robust training.
Linking data from an EHR^b^ into a CDS does not require the use of tools such as SMART^c^ on FHIR^d^.	SMART on FHIR tools allow for interoperability.
Not all well-intended provider-focused CDS use results in improved care.	Test the CDS to determine if it achieves the desired change.

^a^CDS: clinical decision support.

^b^EHR: electronic health record.

^c^SMART: Substitutable Medical Applications and Reusable Technologies.

^d^FHIR: Fast Healthcare Interoperability Resources.

## Appendix

Multimedia Appendix 1 Clinical decision support system (CDS) development overview.

## Abbreviations

API: application programming interface
CDS: clinical decision support
DIODE: De-Implementing Opioid Use and Implementing Optimal Pain Management Following Dental Extractions
EHR: electronic health record
FHIR: Fast Healthcare Interoperability Resources
HL7: Health Level 7
MED-RT: Medication Reference Terminology
NIH: National Institutes of Health
SMART: Substitutable Medical Applications and Reusable Technologies
