# Supplementary material for: Clinical Decision Support to Reduce Opioid Prescriptions for Dental Extractions using SMART on FHIR: Implementation Report
Source: JMIR Med Inform. 2023 Nov 7;11:e45636. doi: 10.2196/45636 (PMC10664010; doi:10.2196/45636)
Supplement: Multimedia Appendix 1 [file medinform_v11i1e45636_app1.pptx]

## Slide 1
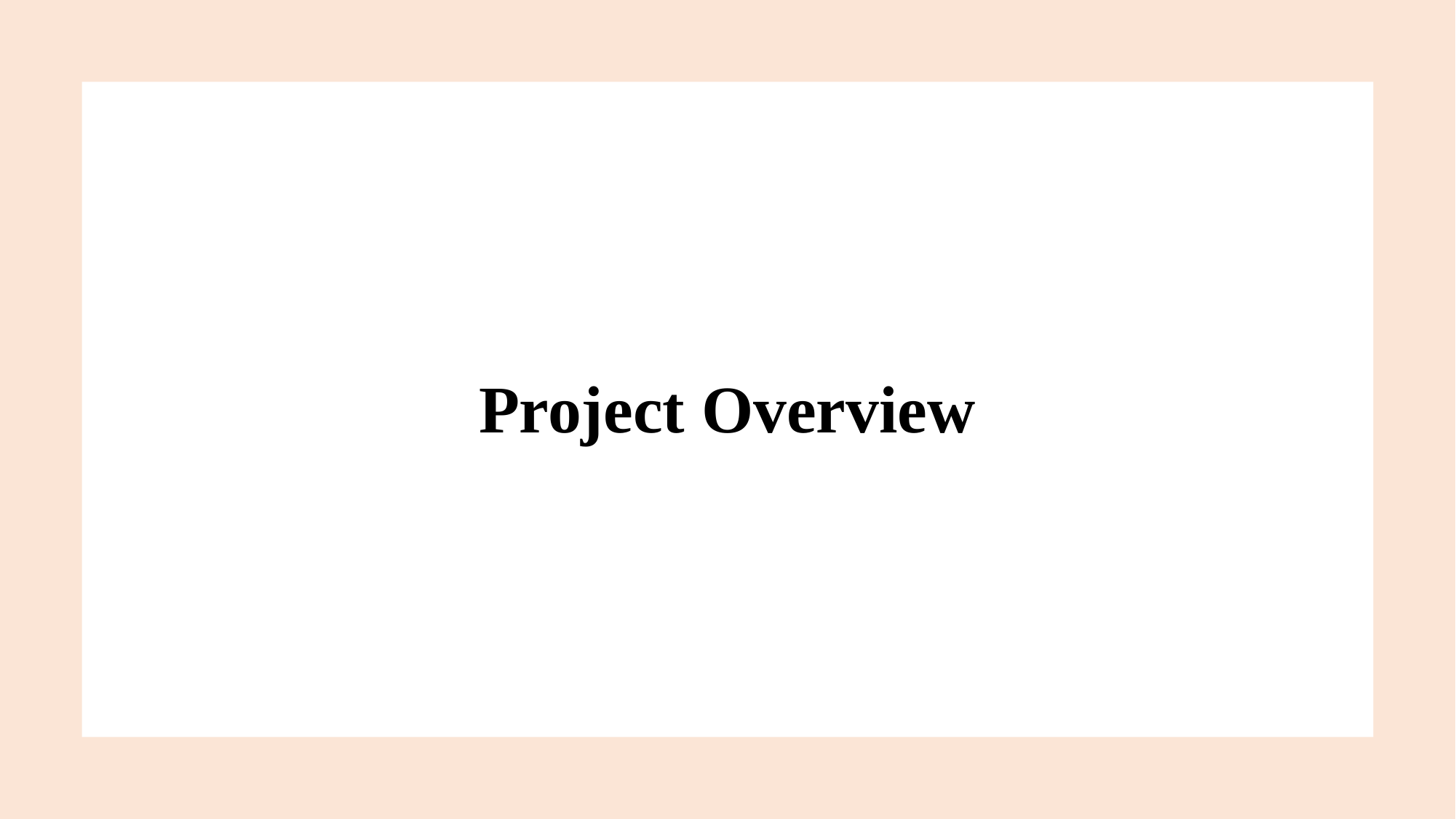

# Project Overview

## Slide 2
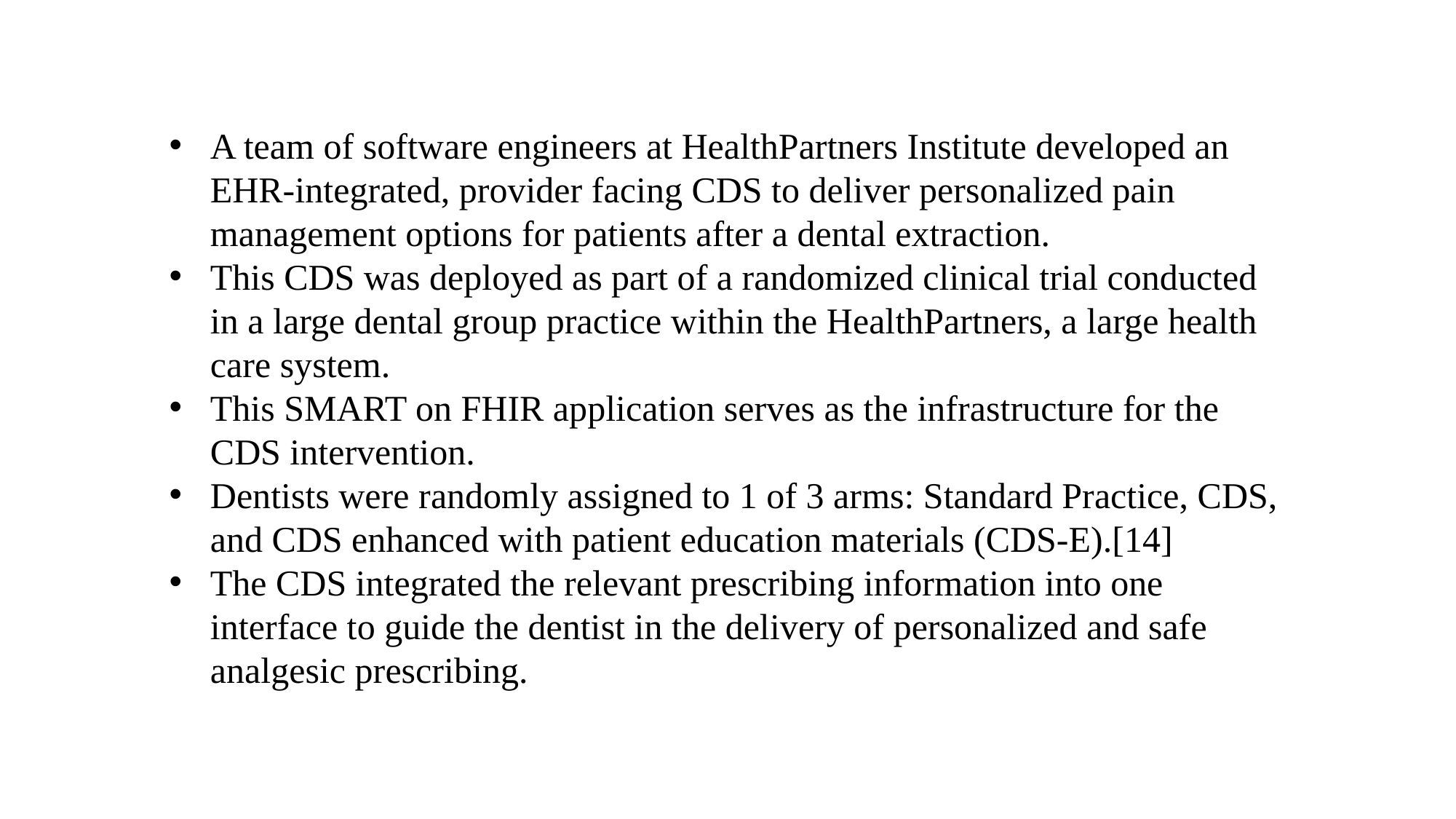

A team of software engineers at HealthPartners Institute developed an EHR-integrated, provider facing CDS to deliver personalized pain management options for patients after a dental extraction.
This CDS was deployed as part of a randomized clinical trial conducted in a large dental group practice within the HealthPartners, a large health care system.
This SMART on FHIR application serves as the infrastructure for the CDS intervention.
Dentists were randomly assigned to 1 of 3 arms: Standard Practice, CDS, and CDS enhanced with patient education materials (CDS-E).[14]
The CDS integrated the relevant prescribing information into one interface to guide the dentist in the delivery of personalized and safe analgesic prescribing.

## Slide 3
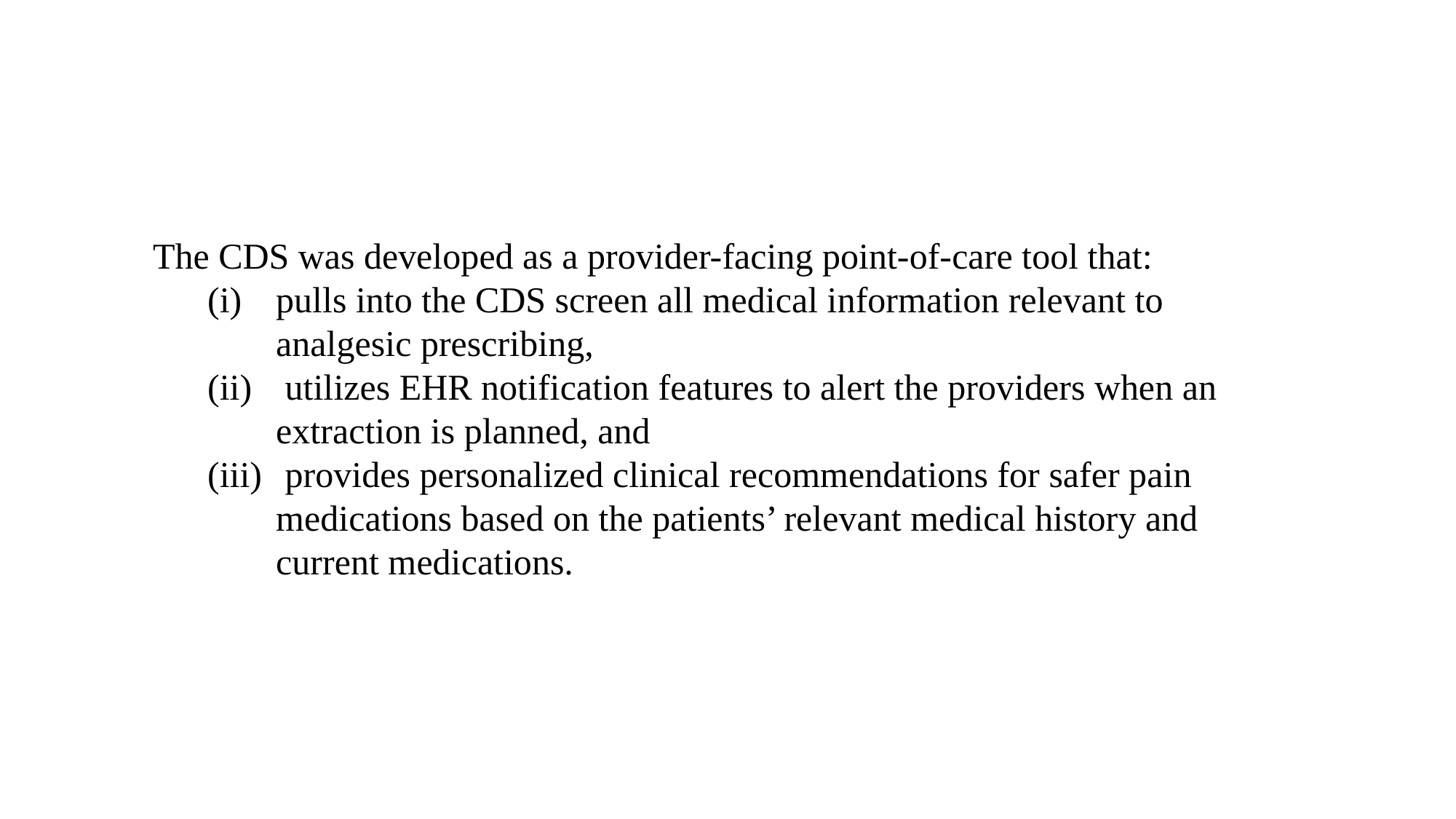

The CDS was developed as a provider-facing point-of-care tool that:
pulls into the CDS screen all medical information relevant to analgesic prescribing,
 utilizes EHR notification features to alert the providers when an extraction is planned, and
 provides personalized clinical recommendations for safer pain medications based on the patients’ relevant medical history and current medications.
